# Use of Albumin-Adjusted Calcium Measurements in Clinical Practice

**DOI:** 10.1001/jamanetworkopen.2024.55251

**Published:** 2025-01-21

**Authors:** Noémie Desgagnés, James A. King, Gregory A. Kline, Isolde Seiden-Long, Alexander A. Leung

**Affiliations:** 1 Department of Medicine, University of Calgary, Calgary, Alberta, Canada; 2 Data and Research Services, Alberta Strategy for Patient-Oriented Research Provincial Research Data Services, Alberta Health Services; 3 Division of Endocrinology and Metabolism, Department of Medicine, University of Calgary, Calgary, Alberta, Canada; 4 Alberta Precision Laboratories and Department of Pathology and Laboratory Medicine, University of Calgary, Calgary, Alberta Canada; 5 Department of Community Health Sciences, University of Calgary, Calgary, Alberta, Canada

## Abstract

**Importance:**

Using albumin-adjusted calcium is commonly recommended for for measuring calcium, but with little empirical evidence to support the practice.

**Objective:**

To assess the correlation between total calcium measurements (with or without adjustment) vs the ionized calcium level as a reference standard.

**Design, Setting, and Participants:**

This was a population-based cross-sectional study in the province of Alberta, Canada, including adults tested for serum total calcium and ionized calcium simultaneously between January 1, 2013, and October 31, 2019. Statistical analysis was performed from March 2023 to October 2024.

**Main Measures and Outcomes:**

The correlation between unadjusted and adjusted total calcium measurements (using 10 formulas) and the ionized calcium level was evaluated, along with the potential association with the classification of calcium status.

**Results:**

Among 22 658 patients included, 11 889 (52.5%) were female and 10 769 (47.5%) were male; the median (IQR) age was 60 (47-72) years. The unadjusted total calcium (*R*^2^ = 71.7%; 95% CI, 71.1%-72.2%) had a stronger correlation with ionized calcium than the commonly used simplified Payne formula (ie, total calcium [mmol/L] + 0.02 [40 − albumin (g/L)]) (*R*^2^ = 68.9%; 95% CI, 68.0%-69.6%) and correlated similarly to other formulas (Payne: lowest *R*^2^ = 60.3%; 95% CI, 59.3%-61.3%; and James: highest *R*^2^ = 76.7%; 95% CI, 76.1%-77.3%). When classifying patients into categories of hypocalcemia, normocalcemia, or hypercalcemia, unadjusted total calcium had the best overall agreement (74.5%) with ionized calcium compared with albumin-adjusted calcium using the original Payne and simplified Payne formulas (agreement 63.0% and 58.7%, respectively). Misclassification using the adjustment formulas was worse in the presence of hypoalbuminemia (albumin level <30 g/L).

**Conclusions and Relevance:**

In this cross-sectional study drawn from a contemporaneous population, there appeared to be heavy reliance on adjustment formulas for calcium in clinical practice with little gain but considerable risk of misclassification of true calcium status, especially in the presence of hypoalbuminemia. These results suggest that unadjusted total calcium was the best and most practical alternative to ionized calcium.

## Key Points

**Question:**

How accurate are the calcium adjustment formulas that are used in clinical practice?

**Findings:**

In this cross-sectional study of 22 658 patients, adjusted calcium levels were modestly correlated with ionized calcium; these generally fared no better than unadjusted total calcium alone and had the tendency to systematically underestimate true hypocalcemia and overestimate true hypercalcemia.

**Meaning:**

These results suggest that the unadjusted total calcium is the best and most practical alternative to ionized calcium in most clinical settings.

## Teaser

This cross-sectional study investigates the correlation between measurements of total calcium (with or without adjustment) and ionized calcium level as a reference standard to examine the potential association with disease classification.

## Introduction

Calcium is a tightly regulated mineral that is essential to intracellular signaling, bone mineralization, coagulation, and neuromuscular function. Disorders of calcium metabolism can be associated with considerable morbidity and mortality.^1,2,3^ As such, reliable calcium measurement, accurate classification of calcium status, and timely treatment of hypo- or hypercalcemia, when present, are important.

Approximately half of circulating calcium is bound to serum proteins, principally albumin, and the other half is ionized, but only the ionized fraction is biologically active. Even though ionized calcium is more physiologically relevant, total calcium is more commonly measured because of its greater accessibility and the lower costs for analysis. However, concern has been raised that reliance on total calcium alone may result in misclassification because changes in blood pH and serum protein concentrations can affect calcium-protein binding, and therefore alter the correlation between total calcium and ionized calcium.^4,5^

For decades, adjustment of total calcium has been used clinically as an alternative to ionized calcium^6,7^ with little empirical evidence to support the practice. Indeed, the original correction formula proposed by Payne and colleagues^6^ in 1973 was derived from a single study of 200 patients using a historical laboratory method no longer available today, and never validated against ionized calcium. Furthermore, multiple studies have shown that albumin-adjusted calcium correlates poorly with ionized calcium,^8,9,10,11,12,13,14,15,16^ especially in patients who are critically ill,^17,18,19^ those with kidney failure on dialysis,^20,21,22^ and in geriatric populations.^23,24^ Addressing this, some investigators have attempted to derive new formulas that correlate better with ionized calcium,^8,11,13,16,25,26,27,28^ but with many still concluding that unadjusted total calcium correlates best with ionized calcium in most clinical settings. Although global evidence suggests that most adjustment formulas are not clinically reliable, it is unclear how commonly they are used in practice. Moreover, the generalizability of current evidence remains limited, as prior studies were largely limited to highly selected populations, such as hospitalized patients. Additionally, most of the laboratory data were collected from a single laboratory usually affiliated with a tertiary center.

Addressing this, we conducted a large population-based cross-sectional study to evaluate the correlation between total calcium (with or without adjustment) vs ionized calcium (as a reference standard), to examine the potential association with disease classification (where misdiagnosis could lead to inappropriate downstream investigations and/or treatments), and to describe general calcium ordering patterns in clinical practice.

## Methods

This cross-sectional study was approved by the Conjoint Health Research Ethics Board at the University of Calgary. A waiver of consent was granted for access to personal identifiable health information consistent with the conditions of section 50 of the Health Information Act of Alberta, Canada. Our study was reported according to the Reporting of Studies Conducted Using Observational Routinely-Collected Data (RECORD) guideline.^35^

### Data Sources

We assembled a retrospective, population-based cohort of all adults (≥18 years of age) in the province of Alberta, Canada. Data were obtained from linked administrative databases of Alberta Health Services and Alberta Health, where universal health coverage is provided to 99% of the approximately 4.3 million people. These databases included population registries with details related to patient demographics, the Discharge Abstract Database (DAD), the National Ambulatory Care Reporting System/Ambulatory Care Classification System (NACRS/ACCS), Practitioner Claims (CLM), and laboratory services.^29,30,31^ These data have been used in numerous studies and are considered to be high quality and comprehensive.^32,33^

### Study Population

**Figure 1.**
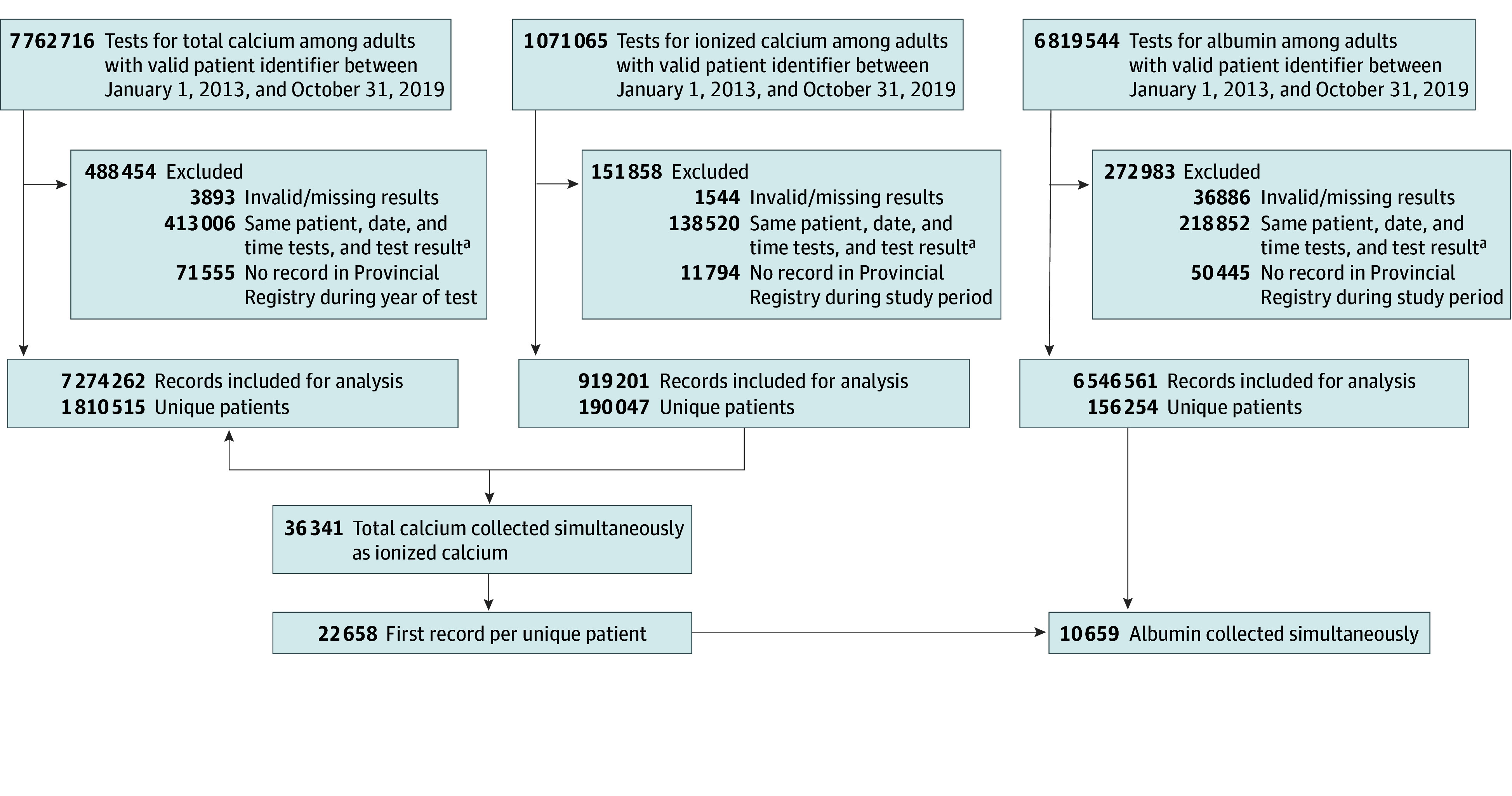
Flow Diagram for Inclusion and Exclusion of Relevant Tests ^a^Or 1 sample analyzed more than once (mean value taken).

We identified adults (≥18 years of age) who were tested simultaneously (ie, same date and time) for serum total calcium and ionized calcium between January 1, 2013, and October 31, 2019. Records were excluded if there was an invalid or missing patient identifier and/or test result (Figure 1). Baseline age and sex were retrieved from the health insurance registry. Results were stratified according to the collection location (inpatient/emergency department [ED] vs outpatient). Laboratory information systems were used to retrieve measurements of total calcium and ionized calcium for all patients; when available, serum albumin, serum creatinine, estimated glomerular filtration rate, and blood pH were also obtained. Prior comorbidities (congestive heart failure, cirrhosis, cancer [single site or metastatic]) were identified with *International Classification of Diseases, Ninth Revision (ICD-9)* and *ICD-10* codes based on previously validated definitions.^30,31,34^ These records were linked per patient prior to their index calcium tests and went as far back as April 1, 2002.

### Laboratory Methods

Serum total calcium, albumin, creatinine/estimated glomerular filtration rate (eGFR), ionized calcium and pH were measured with a variety of instruments and methods by Alberta Precision Laboratories. This organization is the sole provider of laboratory services in the province of Alberta (eTable 1 in Supplement 1).

### Correlation Between Total Calcium, Adjusted Calcium, and Ionized Calcium

We examined 10 adjustment formulas (eTable 2 in Supplement 1) that have been proposed for clinical practice, including the commonly used simplified Payne formula: adjusted calcium (mmol/L) = total calcium (mmol/L) + 0.02 [40 − albumin (g/L)].^6,7^ Formulas were converted, if necessary, to SI units for standardization. All of the formulas that were examined were originally developed to adjust for total calcium (ie, by accounting for differences in protein binding), except for those developed by Pekar^8^ and Antonio,^36^ which were designed to directly estimate ionized calcium. To assess the association with albumin status, we performed a subanalysis in patients with low serum albumin (<30 g/L) vs normal serum albumin (30-50 g/L). We conducted a Bland-Altman analysis and applied a linear transformation to standardize total calcium and ionized calcium onto the same scale (using a scaling factor [m] of [2.60 − 2.10]/[1.35 − 1.15] = 2.5 and an offset [b] of 2.10 − [2.5 × 1.15] = −0.775).

### Frequency of Laboratory Measurements

To assess physician ordering patterns, we determined the frequency that serum calcium, ionized calcium, and serum albumin were measured simultaneously (collected at the same date and time). We described variations in these ordering patterns based on individual-level (eg, age, sex, and kidney function) and system-level characteristics (eg, clinical setting) over time.

### Statistical Analysis

Descriptive statistics were reported for baseline demographics, comorbidities, and laboratory measures. Recognizing that some patients may have multiple calcium measurements during the study period, we only considered the first total calcium per patient. For each total calcium measurement, we calculated the adjusted calcium using the 10 aforementioned formulas. The unadjusted and adjusted calcium values were then compared with the simultaneously collected ionized calcium (representing the reference standard). Overall agreement for each pair of comparisons was assessed using linear regression methods and correlation coefficients (and 95% CIs) were reported. We then assessed the potential association of using total calcium or a correction formula with the classification of clinical calcium status, compared with ionized calcium. Based on the ionized calcium value, we classified each patient according to the following categories: hypocalcemia (<1.15 mmol/L), normocalcemia (1.15-1.35 mmol/L), or hypercalcemia (>1.35 mmol/L). We then repeated the same categorization using the matching unadjusted total calcium (hypocalcemia <2.10 mmol/L; normocalcemia 2.10-2.60 mmol/L; hypercalcemia >2.60 mmol/L) and the adjusted calcium (according to the Payne formula and the simplified Payne formula) to assess the overall agreement. These were then compared using a reclassification matrix. Statistical analyses were performed using SAS version 9.4 (SAS Institute) and RStudio version 3.5.2 (R Foundation for Statistical Computing) from March 2023 to October 2024.

## Results

### Baseline Demographics

Our study included a total of 22 658 patients (Table 1), of whom 11 889 (52.5%) were female and 10 769 (47.5%) were male, with a median (IQR) age of 60 (47-72) years. Congestive heart failure was present in 3734 (16.5%), malignant neoplasm in 2631 (11.6%), and cirrhosis in 1134 (5.0%); 17 157 patients (75.7%) had a serum creatinine measured during the study period and 11 205 (49.5%) had an eGFR of at least 60 mL/min/1.73 m^2^; and 11 205 (50.6%) were located in Calgary, representing the largest city in the province.^37^

**Table 1.** Patient Characteristics

Characteristic	Patients, No. (%)									
	All	Clinical setting				Zone				
		Inpatient	ED	Outpatient	Other/unknown	North	Edmonton	Central	Calgary	South
Individual patient, No.^a^	22 658	9671 (42.7)	7032 (31.0)	4766 (21.0)	1189 (5.3)	2462 (10.9)	4437 (19.6)	1881 (8.3)	11 465 (50.6)	2409 (10.6)
Age, median (IQR), y	60 (47-72)	62 (50-73)	61 (44-76)	56 (44-66)	59 (47-69)	58 (44-70)	60 (47-70)	60 (48-69)	61 (47-73)	63 (51-75)
Sex										
Female	11 889 (52.5)	4596 (47.5)	3332 (47.5)	3170 (66.5)	791 (66.5)	1240 (50.4)	2370 (53.4)	997 (53.0)	6028 (52.6)	1253 (52.0)
Male	10 769 (47.5)	5075 (52.5)	3700 (52.5)	1596 (33.5)	398 (33.5)	1222 (49.6)	2067 (46.6)	884 (47.0)	5437 (47.4)	1156 (48.0)
Comorbidities										
Congestive heart failure	3734 (16.5)	2127 (22.0)	1231 (17.5)	290 (6.1)	86 (7.2)	399 (16.2)	688 (15.5)	310 (16.5)	1745 (15.2)	591 (24.5)
Cirrhosis	1135 (5.0)	583 (6.0)	453 (6.4)	79 (1.7)	20 (1.7)	87 (3.5)	193 (4.4)	95 (5.1)	647 (5.6)	113 (4.7)
Cancer (single site)	2631 (11.6)	1355 (14.0)	828 (11.8)	344 (7.2)	104 (8.8)	250 (10.2)	527 (11.9)	199 (10.6)	1339 (11.7)	316 (13.1)
Cancer (metastatic)	1540 (6.8)	846 (8.8)	464 (6.6)	151 (3.2)	79 (6.6)	149 (6.1)	336 (7.6)	128 (6.8)	740 (6.5)	187 (7.8)
Ionized calcium, median (IQR), mmoL/L	1.17 (1.11-1.25)	1.13 (1.07-1.20)	1.16 (1.11-1.21)	1.26 (1.20-1.33)	1.26 (1.18-1.33)	1.15 (1.10-1.20)	1.14 (1.08-1.21)	1.17 (1.08-1.29)	1.19 (1.12-1.27)	1.16 (1.11-1.23)
Total calcium, median (IQR), mmol/L	2.23 (2.05-2.38)	2.08 (1.94-2.23)	2.24 (2.12-2.35)	2.41 (2.31-2.54)	2.33 (2.23-2.47)	2.16 (2.00-2.29)	2.17 (2.01-2.33)	2.19 (2.00-2.35)	2.28 (2.11-2.42)	2.17 (2.03-2.31)
Albumin	10 659 (47.0)	3835	3945	2336	543	1077	1432	863	6190	1095
Albumin, median (IQR), g/L	34 (27-38)	27 (22-32)	34 (29-38)	39 (36-42)	38 (34-40)	33 (26-38)	34 (28-41)	31 (24-38)	34 (28-38)	30 (24-36)
eGFR, mL/min/1.73 m^2^	17 157 (75.7)	7618 (78.8)	6326 (90.0)	2630 (55.2)	583 (49.0)	1963 (79.7)	3102 (69.9)	1282 (68.2)	8809 (76.8)	1997 (82.9)
≥60	11 205 (49.5)	4571 (47.3)	4150 (59.0)	2065 (43.3)	419 (35.2)	1303 (52.9)	2194 (49.5)	786 (41.8)	5790 (50.5)	1129 (46.9)
30-59	3424 (15.1)	1560 (16.1)	1335 (19.0)	418 (8.8)	111 (9.3)	382 (15.5)	474 (10.7)	255 (13.6)	1852 (16.2)	460 (19.1)
15-29	1392 (6.1)	828 (8.6)	474 (6.7)	60 (1.3)	30 (2.5)	143 (5.8)	223 (5.0)	156 (8.3)	644 (5.6)	226 (9.4)
<15	1136 (5.0)	659 (6.8)	367 (5.2)	87 (1.8)	23 (1.9)	135 (5.5)	211 (4.8)	85 (4.5)	523 (4.6)	182 (7.6)

Abbreviations: ED, emergency department; eGFR, estimated glomerular filtration rate.

SI unit conversions: To convert ionized calcium to mg/dL, divide by 0.25; total calcium to mg/dL, divide by 0.25; albumin to g/dL, divide by 10.

^a^ First simultaneous measurement of total calcium and ionized calcium, per patient.

### Correlation Between Total Calcium, Adjusted Calcium, and Ionized Calcium

**Figure 2.**
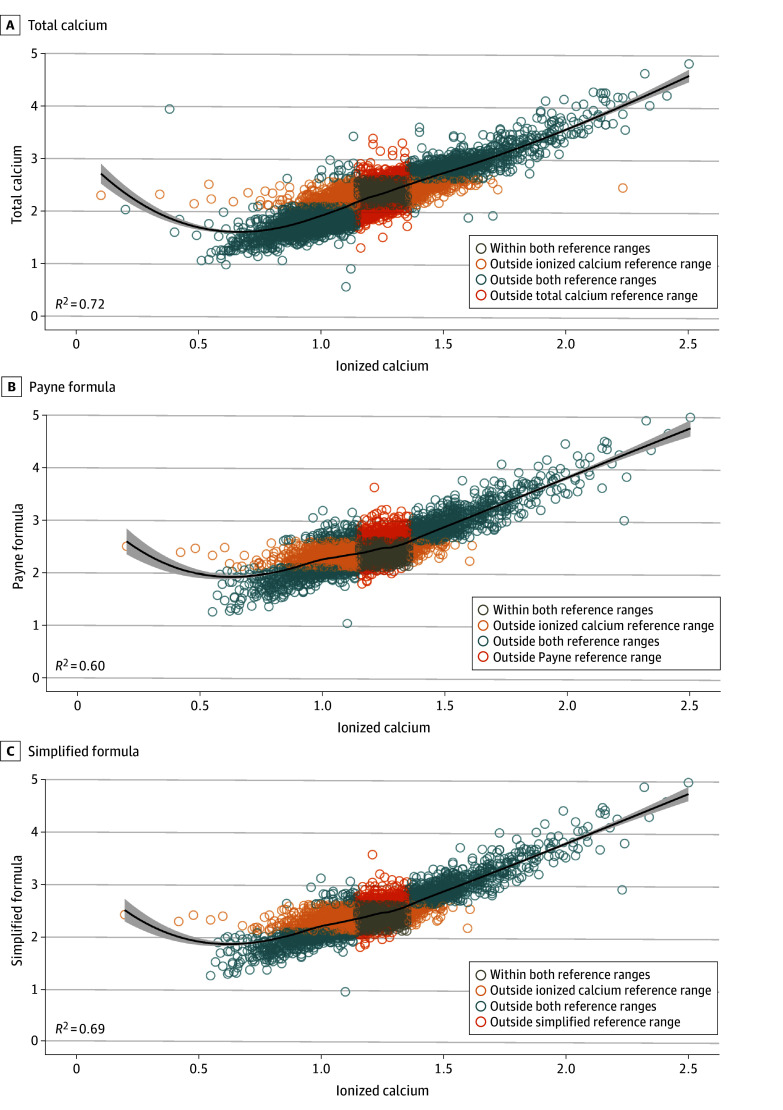
Correlation Between Total Calcium and Adjusted Calcium Measurements vs Ionized Calcium Levels Figure shows the correlation between total calcium and adjusted calcium (total calcium [A]; Payne formula [B]; simplified formula [C]) vs ionized calcium with corresponding correlation coefficient.

Unadjusted total calcium (*R*^2^ = 71.7%; 95% CI, 71.1%-72.2%) had a stronger correlation with ionized calcium than the commonly used simplified Payne formula: total calcium (mmol/L) + 0.02 [40 − albumin (g/L)] (*R*^2^ = 68.9%; 95% CI, 68.0%-69.6%) (Figure 2). The original Payne formula had the weakest correlation (*R*^2^ = 60.3%; 95% CI, 59.3%-61.3%) whereas the James formula, which uses a different correction coefficient, had the best overall correlation (*R*^2^ = 76.7%; 95% CI, 76.1%-77.3%) (Figure 2; eFigure 1 in Supplement 1). The Pekar formula had a slightly better correlation (*R*^2^ = 74.1%; 95% CI, 73.3%-74.8%) with ionized calcium than unadjusted total calcium, but was also the most complex formula, adjusting for albumin, kidney function, and pH (eFigure 2 in Supplement 1). The remaining correction formulas (Berry, Thode, Antonio A and B) had a weaker correlation with ionized calcium compared with unadjusted total calcium (eFigures 1 and 2, eTable 3 in Supplement 1). The findings were similar when the analysis was repeated in patients with hypoalbuminemia (albumin level <30 g/L) (eTable 4 in Supplement 1). The Bland-Altman plots revealed a negative bias for the Payne and simplified formulas, especially at lower calcium levels (<2.10 mmol/L) (eFigure 3 in Supplement 1). A similar pattern was present for the other formulas that adjusted for total calcium (Orrell, Berry, Thode, and James) (eFigure 4 in Supplement 1) and those estimating ionized calcium (Antonio and Pekar), with a negative bias observed at lower calcium levels (eFigure 5 in Supplement 1).

### Classification of Calcium Status

When implemented, adjusted calcium levels (using the original Payne formula and the simplified Payne formula) commonly resulted in large discordances in calcium status compared with ionized calcium (classification agreement 58.7% and 63.0%, respectively). Using the Payne formula, patients were misclassified by 1 category in 40.0% of cases (eg, from hypocalcemia to normocalcemia) and by 2 categories in 1.3% (eg, from hypocalcemia to hypercalcemia). Findings were similar overall when the simplified correction formula was used with misclassification by 1 category in 36.6% of cases and by 2 categories in 0.5%. In contrast, the unadjusted total calcium had the best overall agreement with ionized calcium (74.5%). Patients were misclassified by 1 category in 25.3% of cases and by 2 categories in 0.1% (Table 2).

**Table 2.** Classification of Calcium Status by Total Calcium and Ionized Calcium

	Classification by ionized calcium, No. (% of total)			
	Hypocalcemia	Normocalcemia	Hypercalcemia	Total
**Classification by adjusted calcium using Payne formula**				
Hypocalcemia	532 (5.0)	46 (0.4)	0	578 (5.4)
Normocalcemia	3233 (30.3)	4750 (44.6)	188 (1.8)	8171 (76.8)
Hypercalcemia	133 (1.3)	799 (7.5)	978 (9.2)	1910 (17.9)
Total	3898 (36.6)	5595 (52.5)	1166 (10.9)	10 659 (100)
Observed agreement	NA	NA	NA	6260 (58.7)
**Classification by adjusted calcium using simplified formula**				
Hypocalcemia	674 (6.3)	32 (0.3)	0	706 (6.7)
Normocalcemia	3175 (29.8)	5099 (47.8)	220 (2.1)	8494 (79.7)
Hypercalcemia	49 (0.5)	464 (4.4)	946 (8.9)	1459 (13.7)
Total	3898 (36.6)	5595 (52.5)	1166 (10.9)	10 659 (100)
Observed agreement	NA	NA	NA	6719 (63.0)
**Classification by total calcium**				
Hypocalcemia	5653 (25.0)	1202 (5.3)	6 (0.0)	6861 (30.3)
Normocalcemia	3476 (15.3)	9865 (43.5)	753 (3.3)	14 094 (62.2)
Hypercalcemia	23 (0.1)	313 (1.4)	1367 (6.0)	1703 (7.5)
Total	9152 (40.4)	11 380 (50.2)	2126 (9.4)	22 658 (100)
Observed agreement	NA	NA	NA	16 885 (74.5)

Abbreviation: NA, not applicable.

The most common type of misclassification was when true hypocalcemia (based on ionized calcium) was reclassified to normocalcemia (in 30.3% of patients using the original Payne formula; 29.8% using the simplified formula; and 15.3% based on total calcium alone). Misclassification with the adjustment formulas was much higher in the presence of hypoalbuminemia (ie, albumin level <30 g/L; when adjustment formulas are most likely to be used in clinical practice) compared with when albumin was normal (ie, 30-50 g/L) (eTables 5 and 6 in Supplement 1). Inappropriate reclassification of true normocalcemia to hypercalcemia was also relatively common (in 7.5% of patients using the original Payne formula; 4.4% using the simplified formula; and 1.4% based on total calcium alone) (Table 2).

### Laboratory Ordering Patterns

When looking at all tests collected during the study period (and allowing for more than 1 measurement per patient), there were 7 274 442 serum total calcium tests, 6 546 561 serum albumin tests, and 919 381 ionized calcium tests (eFigure 6 in Supplement 1). The majority of total calcium measurements (54.8%; n = 3 990 000) were ordered with a serum albumin, suggesting that albumin-adjusted calcium corrections were commonly used in clinical practice; less than half (44.9%; n = 3 265 467) of total calcium measurements were ordered alone, and very rarely (0.5%; n = 36 521) total calcium was ordered with an ionized calcium. In most cases when a serum albumin was measured (60.9%; n = 3 990 000), it was with a total calcium, suggesting that these were likely ordered with the intention of being used for calculating an adjusted calcium.

Ordering patterns were similar, irrespective of patient age, sex, or kidney function (eTable 7 in Supplement 1) and over time (eFigure 7 in Supplement 1). Ionized calcium was mostly ordered in inpatient and emergency department settings (94.7%; n = 870 679) compared with outpatient settings (4.4%; n = 40 370), whereas approximately half of the orders for combined total calcium and albumin were from the outpatient setting (55.1%; n = 2 197 193) (eTable 8 in Supplement 1).

## Discussion

In this cross-sectional study, we found that total calcium and serum albumin were commonly ordered together. In a population of 4.3 million people over approximately 7 years, there were more than 7 million measurements of total calcium, with approximately 4 million paired with serum albumin, suggesting a heavy reliance on albumin-adjusted calcium. Of concern, albumin-adjusted calcium only correlated modestly with ionized calcium and generally fared no better than (unadjusted) total calcium alone, but rather had the tendency to systematically underestimate true hypocalcemia, particularly in the presence of low serum albumin (ie, a scenario where adjustment formulas are likely to be relied on the most but paradoxically where they are least reliable). Therefore, the additional cost and inconvenience of manually calculating an albumin-adjusted calcium are difficult to justify, especially given the excess risk of misclassifying calcium status, which can lead to incorrect downstream treatment decisions. Instead, our findings suggested that total calcium alone was the best available alternative to directly measuring ionized calcium, against common practice.

Our study is consistent with and extends the findings of previous reports. For decades, adjustment of total calcium has been entrenched in clinical practice to account for states of altered protein-binding, as an alternative to ionized calcium. However, these adjustment formulas have been repeatedly shown to be unreliable.^9,10,11,17,18,20,21,23,36,38^ As previously mentioned, the original correction formula proposed by Payne and colleagues was derived from a single laboratory using an albumin measurement method no longer available today, and never validated against ionized calcium.^6^ A modification was subsequently published in 1977, as a practical simplification, but not derived or validated in any patient cohort either.^7^ These correction formulas assumed that binding between calcium and albumin remained constant; but in reality, the calcium-albumin binding ratio has been shown to increase with hypoalbuminemia.^39^ As such, albumin-adjusted calcium has proven to be insensitive to hypocalcemia in hypoalbuminemic states.^9,10,11,17,18,20,21,23,36,38^ Moreover, the accuracy of albumin measurement itself is reduced in the setting of kidney failure.^40^ Acknowledging these limitations, new formulas have been proposed (mostly accounting for differences in calcium-albumin binding and kidney function), but for the most part, none of these have significantly improved upon using total calcium alone.^27,38,41^ Of note, one of the best-performing correction formulas was proposed by Pekar and colleagues,^8^ designed to directly estimate ionized calcium, and this showed marginal improvement in the overall correlation between the estimated and actual ionized calcium (*R*^2^, 74.1%) compared with using total calcium alone (*R*^2^, 71.7%), but the formula requires 4 laboratory measurements (ie, total calcium, albumin, creatinine, and pH) rather than 1, and is therefore more costly and cumbersome to implement than simply measuring ionized calcium itself (ie, measurement of blood pH would require an analyzer that already directly measures ionized calcium).^8,42,43^ Finally, it appears that locally derived formulas may offer incremental improvement in accuracy. For instance, the formula proposed by James and colleagues^38^ was derived in the Calgary Health Region and performed the best in our study. However, the modest improvement in correlation shown in this study is unlikely to outweigh the complexity required to routinely implement this labor-intensive strategy of local derivation and validation in other jurisdictions.

In light of these factors, we suggest that unadjusted calcium remains the best and most practical alternative to ionized calcium in most clinical settings. Clinicians should be thoughtful about ordering ionized calcium instead of total calcium. Ionized calcium should be considered to inform on acute management of hypocalcemia or hypercalcemia in hospitalized patients, especially in the critically ill. It should also be considered in patients with preexisting calcium disorders (eg, hypo- or hyperparathyroidism) when the total calcium does not reflect a patient’s symptoms or clinical status.^44,45^ This is particularly important in the current health care climate, where there is emphasis on the reduction of unnecessary testing.

### Strengths and Limitations

To our knowledge, this is the largest study to date assessing the epidemiology of calcium measurements and its correlation with ionized calcium as the reference standard. We also assessed laboratory ordering patterns, rarely reported in previous studies. Our data are based on a large population with comprehensive capture of all clinical encounters using high-quality linked health administrative and laboratory databases. All laboratory measurements were performed as part of routine clinical care, reducing the risk of selection bias. In comparison to previous studies, which have mostly been conducted at a single tertiary institution with a centralized laboratory, the present study included multiple sites (inpatient and outpatient, urban and rural, academic and community) with variety of laboratory platforms, which increases the generalizability of the findings.

Despite these strengths, our study was also subject to limitations. First, missing data could have affected our results. However, only 4000 records from approximately 7.7 million total calcium tests (0.05%) were excluded due to missing results. As such, the overall effect of missing data on our final conclusions is likely negligible. Second, the laboratory measurements were not performed in a centralized laboratory according to stringent research standards, but rather as part of routine clinical care across multiple platforms. Consequently, total calcium, ionized calcium, albumin, pH, and creatinine were measured using a variety of possible instruments and techniques. Even so, our pragmatic analysis can be construed as a strength because it provides data that reflect practice patterns not otherwise captured by highly selective single-center studies. With regard to measurement error, all tests were conducted using calibrated instruments meeting laboratory clinical standards. Third, we did not examine subgroups according to the presence of acid-base status, age, or kidney function because our overarching goal was to assess a large population with results that would be applicable in most clinical settings. However, numerous previous studies have already consistently shown a poor correlation between adjusted-calcium and ionized calcium in these settings.^9,10,11,17,18,20,21,23,36,38^ Additionally, we were not able to prove the intent of the clinicians who ordered the tests. We inferred that concurrent total calcium and serum albumin were likely used to calculate an albumin-adjusted calcium, but we acknowledge that there may have been other reasons that a serum albumin could have been requested. However, clinical indications for measuring serum albumin are few, so it is implausible that many of these albumin measurements were ordered for a different purpose.

## Conclusions

In this large, province-based cross-sectional study, the adjustment of serum calcium was still commonly used in clinical practice but did not outperform measuring unadjusted total calcium, especially in the setting of hypoalbuminemia. Our results suggest that clinicians should reconsider usage of adjustment formulas in routine practice as these are frequently unreliable, present an unnecessary step and cost, and are prone to misclassification with little to no gain.

## Notes

Supplement 1. **eTable 1.** List of Laboratory Methods Across the Province of Alberta **eTable 2.** Adjustment Formulas for Calcium **eTable 3.** Correlation Between Ionized Calcium and Other Adjusted Calcium Formulas **eTable 4.** Correlation Between Ionized Calcium and Other Adjusted Calcium Formulas **eTable 5.** Classification of Calcium Status by Total Calcium and Ionized Calcium Among Patients With Albumin <30 g/L **eTable 6.** Classification of Calcium Status by Total Calcium and Ionized Calcium Among Patients With Albumin Between 30 and 50 g/L **eTable 7.** Ordering Patterns of Calcium and Related Tests According to Patient-Level Characteristics **eTable 8.** Ordering Patterns of Calcium and Related Tests According to System-Level Characteristics **eFigure 1.** Correlation Between Total Calcium and “Adjusted” Calcium (A = Orrell; B = Berry; C = Thode; D = James) vs. Ionized Calcium With Corresponding Correlation Coefficient **eFigure 2.** Correlation Between Total Calcium and “Adjusted” Calcium (A = Antonio [A]; B = Antonio [B]; C = Antonio [C]; D = Pekar) vs. Ionized Calcium With Corresponding Correlation Coefficient **eFigure 3.** Bland-Altman Plot for Total Calcium (A), Payne (B), and Simplified (C) vs Ionized Calcium **eFigure 4.** Bland-Altman Plot for Orrell (A), Berry (B), Thode (C), and James vs Ionized Calcium **eFigure 5.** Bland-Altman Plot for Antonio A (A), Antonio B (B), Antonio C (C), and Pekar vs Ionized Calcium **eFigure 6.** Number of Total Calcium and Related Tests Ordered **eFigure 7.** Ordering Pattern of Total Calcium, Albumin, and Ionized Calcium Over Time

Supplement 2.
Data Sharing Statement
