# Errors in Results and Table 1

**DOI:** 10.1001/jamanetworkopen.2025.6345

**Published:** 2025-03-18

In the Original Investigation titled “Use of Albumin-Adjusted Calcium Measurements in Clinical Practice,”^1^ published January 21, 2025, there were errors in the Results and Table 1. The unit of eGFR should have been listed as “mL/min/1.73 m^2^” in the Results and Table 1. This article has been corrected.^1^
