# Supplementary material for: Use of Albumin-Adjusted Calcium Measurements in Clinical Practice
Source: JAMA Netw Open. 2025 Jan 21;8(1):e2455251. doi: 10.1001/jamanetworkopen.2024.55251 (PMC11751745; doi:10.1001/jamanetworkopen.2024.55251)
Supplement: Supplement 1. — eTable 1. List of Laboratory Methods Across the Province of Alberta eTable 2. Adjustment Formulas for Calcium eTable 3. Correlation Between Ionized Calcium and Other Adjusted Calcium Formulas eTable 4. Correlation Between Ionized Calcium and Other Adjusted Calcium Formulas eTable 5. Classification of Calcium Status by Total Calcium and Ionized Calcium Among Patients With Albumin <30 g/L eTable 6. Classification of Calcium Status by Total Calcium and Ionized Calcium Among Patients With Albumin Between 30 and 50 g/L eTable 7. Ordering Patterns of Calcium and Related Tests According to Patient-Level Characteristics eTable 8. Ordering Patterns of Calcium and Related Tests According to System-Level Characteristics eFigure 1. Correlation Between Total Calcium and “Adjusted” Calcium (A = Orrell; B = Berry; C = Thode; D = James) vs. Ionized Calcium With Corresponding Correlation Coefficient eFigure 2. Correlation Between Total Calcium and “Adjusted” Calcium (A = Antonio [A]; B = Antonio [B]; C = Antonio [C]; D = Pekar) vs. Ionized Calcium With Corresponding Correlation Coefficient eFigure 3. Bland-Altman Plot for Total Calcium (A), Payne (B), and Simplified (C) vs Ionized Calcium eFigure 4. Bland-Altman Plot for Orrell (A), Berry (B), Thode (C), and James vs Ionized Calcium eFigure 5. Bland-Altman Plot for Antonio A (A), Antonio B (B), Antonio C (C), and Pekar vs Ionized Calcium eFigure 6. Number of Total Calcium and Related Tests Ordered eFigure 7. Ordering Pattern of Total Calcium, Albumin, and Ionized Calcium Over Time [file jamanetwopen-e2455251-s001.pdf]

Supplemental Online Content

Desgagnés N, King JA, Kline GA, Seiden-Long I, Leung AA. Use of albumin-adjusted calcium in clinical practice. *JAMA Netw Open.* 2025;8(1):e2455251. doi:10.1001/jamanetworkopen.2024.55251

eTable 1. List of Laboratory Methods Across the Province of Alberta

eTable 2. Adjustment Formulas for Calcium

eTable 3. Correlation Between Ionized Calcium and Other Adjusted Calcium Formulas

eTable 4. Correlation Between Ionized Calcium and Other Adjusted Calcium Formulas

eTable 5. Classification of Calcium Status by Total Calcium and Ionized Calcium Among Patients With Albumin <30 g/L

eTable 6. Classification of Calcium Status by Total Calcium and Ionized Calcium Among Patients With Albumin Between 30 and 50 g/L

eTable 7. Ordering Patterns of Calcium and Related Tests According to Patient-Level Characteristics

eTable 8. Ordering Patterns of Calcium and Related Tests According to System-Level Characteristics

eFigure 1. Correlation Between Total Calcium and “Adjusted” Calcium (A = Orrell; B = Berry; C = Thode; D = James) vs. Ionized Calcium With Corresponding Correlation Coefficient

eFigure 2. Correlation Between Total Calcium and “Adjusted” Calcium (A = Antonio [A]; B = Antonio [B]; C = Antonio [C]; D = Pekar) vs. Ionized Calcium With Corresponding Correlation Coefficient

eFigure 3. Bland-Altman Plot for Total Calcium (A), Payne (B), and Simplified (C) vs Ionized Calcium

eFigure 4. Bland-Altman Plot for Orrell (A), Berry (B), Thode (C), and James vs Ionized Calcium

eFigure 5. Bland-Altman Plot for Antonio A (A), Antonio B (B), Antonio C (C), and Pekar vs Ionized Calcium

eFigure 6. Number of Total Calcium and Related Tests Ordered

eFigure 7. Ordering Pattern of Total Calcium, Albumin, and Ionized Calcium Over Time

This supplemental material has been provided by the authors to give readers additional information about their work.

© 2025 Desgagnés N et al. *JAMA Network Open*.

eTable 1. List of laboratory methods across the province of Alberta

Test	Manufacturer	Method
Creatinine	Roche Cobas, QuidelOrtho Vitros	Enzymatic
	Siemens Vista and EXL, Beckman DxC, Siemens Atellica	Jaffe (alkaline picrate)
Total Calcium	Roche Cobas	Colorimetric (NM-BAPTA)
	Beckman DxC	Indirect Potentiometric Electrode
	QuidelOrtho Vitros	Colorimetric (Arsenazo III)
	Siemens Vista, EXL, and Atellica	Colorimetric (o-Cresolphthalein Complexone)
Albumin	Beckman DxC, Siemens Atellica, EXL and Vista, Roche Cobas	Colorimetric (Bromocresol Purple)
	QuidelOrtho Vitros	Colorimetric (Bromocresol Green)
pH	Werfen GEM premier, Abbott iSTAT, Radiometer ABL	Direct Potentiometric Electrode
Ionized Calcium	Werfen GEM premier, Abbott iSTAT, Radiometer ABL	Direct Potentiometric Electrode

© 2025 Desgagnés N et al. *JAMA Network Open*.

eTable 2. Adjustment formulas for calcium

Formulas		Variables required (SI units)
Total calcium		Total calcium (mmol/L)
Payne[6]	total calcium (mg/100mL)[a] – albumin (g/100mL)[b] + 4	Total calcium (mmol/L)<br>Albumin (g/L)
Simplified[7]	total calcium (mmol/L) + 0.02 [40 – albumin (g/L)]	Total calcium(mmol/L)<br>Albumin(g/L)
Orrell[46]	total calcium (mmol/L) +0.0177 [34 – albumin (g/L)]	Total calcium (mmol/L)<br>Albumin (g/L)
Berry[47]	total calcium (mmol/L) +0.0225 [46 – albumin (g/L)]	Total calcium(mmol/L)<br>Albumin (g/L)
Thode[48]	total calcium (mmol/L) x [2.7 / (1.7 + (albumin (g/L) / 42 (g/L)]	Total calcium (mmol/L)<br>Albumin (g/L)
James[37]	total Ca (mmol/L) +0.012 [39.9 – albumin (g/L)]	Total calcium<br>Albumin (g/L)
Antonio (A)[38]	0.815 x total calcium$^{0.5}$ (mmol/L)	Total calcium (mmol/L)
Antonio (B)[38]	0.826 x total calcium$^{0.5}$ (mmol/L) – 0.023 x renal function (RF)<br>where RF is:<br>• normal function (eGFR > 60 ml/min/1.73m^2) = 0<br>•moderate dysfunction (eGFR 30-59 ml/min/1.73m^2) =1<br>•severe dysfunction (eGFR<30 ml/min/1.73m^2) = 2	Total calcium (mmol/L)<br>eGFR (ml/min/1.73m^2)
Antonio (C)[38]	0.813 x total calcium$^{0.5}$(mmol/L) – 0.006 x Alb$^{0.75}$ (g/L) + 0.079	Total calcium(mmol/L)<br>Albumin(g/L)
Pekar[8]	2.567 – 2.045e -3[albumin (g/L)] – 5.601e - 4 [creatinine (mg/L)[c]] +0.4493[total calcium (mmol/L)] – 0.307[pH]	Total calcium(mmol/L)<br>Albumin(g/L)<br>creatinine (mg/L)<br>pH

© 2025 Desgagnés N et al. *JAMA Network Open*.

eTable 3. Correlation between ionized calcium and other adjusted calcium formulas

Adjusted formula	N	R^2 (95% CI)
Total calcium	22 658	71.7% (71.1, 72.2)*
Payne	10 659	60.3% (59.3, 61.3)*
Simplified	10 659	68.9% (68.0, 69.6)*
Orrell	10 659	72.0% (71.3, 72.7)*
Berry	10 659	64.8% (63.9, 65.7)*
Thode	10 659	65.8% (65.0, 66.7)*
James	10 659	76.7% (76.1, 77.3)*
Antonio (A)	22 658	70.4% (69.9, 70.9)*
Antonio (B)	17 157	66.7% (66.0, 67.3)*
Antonio (C)	10 659	76.1% (75.5, 76.7)*
Pekar	8228	74.1% (73.3, 74.8)*

* $p \leq 0.05$ for the overall R^2 correlation

© 2025 Desgagnés N et al. *JAMA Network Open*.

eTable 4. Correlation between ionized calcium and other adjusted calcium formulas

Adjusted formula	Albumin <30 g/L		Albumin 30-50 g/L	
	N	R^2 (95% CI)	N	R^2 (95% CI)
Total calcium	3617	74.3% (73.1, 75.4)*	7017	75.2% (74.4, 76.0)*
Payne	3617	70.6% (69.3, 71.9)*	7017	70.9% (70.0, 71.9)*
Simplified	3617	73.3% (72.0, 74.4)*	7017	74.1% (73.3, 74.9)*
Orrell	3617	74.2% (73.0, 75.3)*	7017	75.2% (74.4, 76.0)*
Berry	3617	72.1% (70.8, 73.3)*	7017	72.7% (71.8, 73.5)*
Thode	3617	72.1% (70.8, 73.3)*	7017	73.3% (72.4, 74.1)*
James	3617	75.5% (74.3, 76.5)*	7017	76.9% (76.1, 77.6)*
Antonio (A)	3617	72.7% (71.4, 73.9)*	7017	74.4% (73.5, 75.2)*
Antonio (B)	3202	72.2% (70.8, 73.5)*	5691	69.6% (68.5, 70.6)*
Antonio (C)	3617	74.2% (73.0, 75.3)*	7017	76.3% (75.5, 77.0)*
Pekar	2798	72.7% (71.3, 74.0)*	5410	76.0% (75.0, 76.8)*

* $p \leq 0.05$ for the overall R^2 correlation

© 2025 Desgagnés N et al. *JAMA Network Open*.

eTable 5. Classification of calcium status by total calcium and ionized calcium among patients with albumin <30 g/L

**Classification by corrected calcium using Payne formula, n (% of total)**	**Classification by ionized calcium, n (% of total)**			
	Hypocalcemia	Normocalcemia	Hypercalcemia	Total
Hypocalcemia	174 (4.8%)	≤10 (≤0.3%)	0 (0.0%)	174 (4.8%)
Normocalcemia	1634 (45.2%)	909 (25.1%)	≤10 (≤0.3%)	2543 (70.3%)
Hypercalcemia	128 (3.5%)	499 (13.8%)	267 (7.4%)	894 (24.7%)
Total	1936 (53.5%)	1408 (38.9%)	267 (7.4%)	3617 (100%)
**Observed agreement**	**1350 (37.3%)**			
**Classification by corrected calcium using simplified formula, n (% of total)**	**Classification by ionized calcium**			
Hypocalcemia	286 (7.9%)	≤10 (≤0.3%)	0 (0.0%)	286 (7.9%)
Normocalcemia	1606 (44.4%)	1161 (32.1%)	≤10 (≤0.3%)	2767 (76.5%)
Hypercalcemia	44 (1.2%)	245 (6.8%)	263 (7.3%)	552 (15.3%)
Total	1936 (53.5%)	1406 (38.9%)	263 (7.3%)	3617 (100%)
**Observed agreement**	**1710 (47.3%)**			
**Classification by total calcium, n (% of total)**	**Classification by ionized calcium**			
Hypocalcemia	1587 (43.9%)	465 (12.9%)	≤10 (≤0.3%)	2052 (56.7%)
Normocalcemia	347 (9.6%)	936 (25.9%)	110 (3.0%)	1393 (38.5%)
Hypercalcemia	≤10 (≤0.3%)	≤10 (≤0.3%)	161 (4.5%)	161 (4.5%)
Total	1934 (53.4%)	1401 (38.7%)	271 (7.5%)	3617 (100%)
**Observed agreement**	**2684 (74.2%)**			

© 2025 Desgagnés N et al. *JAMA Network Open*.

eTable 6. Classification of calcium status by total calcium and ionized calcium among patients with albumin between 30 and 50 g/L

**Classification by corrected calcium using Payne formula, n (% of total)**	**Classification by ionized calcium, n (% of total)**			
	Hypocalcemia	Normocalcemia	Hypercalcemia	Total
Hypocalcemia	356 (5.1%)	39 (0.6%)	0 (0.0%)	395 (5.6%)
Normocalcemia	1598 (22.8%)	3825 (54.5%)	183 (2.6%)	5606 (79.9%)
Hypercalcemia	≤10 (≤0.1%)	300 (4.3%)	711 (10.1%)	1011 (14.4%)
Total	1954 (27.8%)	4164 (59.3%)	894 (12.7%)	7017 (100%)
**Observed agreement**	**4892 (69.7%)**			
**Classification by corrected calcium using simplified formula, n (% of total)**	**Classification by ionized calcium**			
Hypocalcemia	389 (5.5%)	26 (0.4%)	0 (0.0%)	415 (5.9%)
Normocalcemia	1565 (22.3%)	3919 (55.9%)	211 (3.1%)	5695 (81.2%)
Hypercalcemia	≤10 (≤0.1%)	219 (3.1%)	683 (9.7%)	902 (12.9%)
Total	1954 (27.8%)	4164 (59.3%)	894 (12.7%)	7017 (100%)
**Observed agreement**	**4991 (71.1%)**			
**Classification by total calcium, n (% of total)**	**Classification by ionized calcium**			
Hypocalcemia	758 (10.8%)	122 (1.7%)	≤10 (≤0.1%)	880 (12.5%)
Normocalcemia	1194 (17.0%)	3911 (55.7%)	293 (4.2%)	5398 (76.9%)
Hypercalcemia	≤10 (≤0.1%)	131 (1.9%)	600 (8.9%)	731 (10.4%)
Total	1952 (27.8%)	4164 (59.3%)	893 (12.7%)	7017 (100%)
**Observed agreement**	**5269 (75.1%)**			

© 2025 Desgagnés N et al. *JAMA Network Open*.

eTable 7. Ordering patterns of calcium and related tests according to patient-level characteristics

Orders	All	Age, years			Sex		*eGFR, ml/min/1.73m^2	
		< 40	40-59	> 60	Female	Male	< 60	≥60
**iCa**	919 201	135 397 (14.7%)	264 948 (28.8%)	518 856 (56.5%)	393 104 (42.8%)	526 093 (57.2%)	19 141 (32.4%)	39 880 (67.6%)
**Albumin only**	2 556 561	554 096 (21.7%)	923 785 (36.1%)	1 078 680 (42.2%)	1 385 345 (54.2%)	1 171 176 (45.8%)	21 780 (33.4%)	43 522 (66.6%)
**TCa only**	3 284 262	680 573 (20.7%)	1 089 302 (33.2%)	1 514 387 (46.1%)	1 791 515 (54.6%)	1 492 701 (45.5%)	60 212 (42.4%)	81 728 (57.6%)
**Combined albumin and TCa***	3 990 000	369 402 (16.0%)	1 312 338 (32.9%)	2 038 260 (51.1%)	2 105 925 (52.8%)	1 884 031 (47.2%)	115 143 (51.6%)	107 842 (48.4%)

*Based on measurements collected simultaneously
Abbreviations: eGFR, estimated glomerular filtration rate; iCa, ionized calcium; Tca, total calcium.

© 2025 Desgagnés N et al. *JAMA Network Open*.

eTable 8. Ordering patterns of calcium and related tests according to system-level characteristics

Orders	All	Clinical setting				Zone				
		Inpatient	ED	Outpatient	Other/ unknown	North	Edmonton	Central	Calgary	South
**iCa**	919 201	600 505 (65.3%)	270 174 (29.4%)	40 370 (4.4%)	8152 (0.9%)	45 186 (4.9%)	63 161 (6.9%)	56 192 (6.1%)	639 469 (69.6%)	115 165 (12.5%)
**Albumin only**	2 556 561	333 545 (13.1%)	116 454 (4.6%)	1 660 931 (65.0%)	445 631 (17.4%)	252 971 (9.9%)	693 621 (27.1%)	297 686 (11.6%)	1 132 990 (44.3%)	179 200 (7.0%)
**TCa only**	3 284 262	630 995 (19.2%)	732 916 (22.3%)	1 447 655 (44.1%)	472 696 (14.4%)	421 837 (12.8%)	1 493 083 (45.5%)	332 048 (10.1%)	793 536 (24.2%)	243 662 (7.4%)
**Combined albumin and TCa***	3 990 000	569 615 (14.3%)	267 311 (6.7%)	2 197 193 (55.1%)	955 881 (24.0%)	429 244 (10.8%)	1 281 062 (32.3%)	507 695 (12.8%)	1 480 008 (37.3%)	274 323 (6.9%)

*Based on measurements collected simultaneously
Abbreviations: ED, emergency department; iCa, ionized calcium; TCa, total calcium

© 2025 Desgagnés N et al. *JAMA Network Open*.

eFigure 1: Correlation between total calcium and "adjusted" calcium (A = Orrell; B = Berry; C = Thode; D = James) vs. ionized calcium with corresponding correlation coefficient

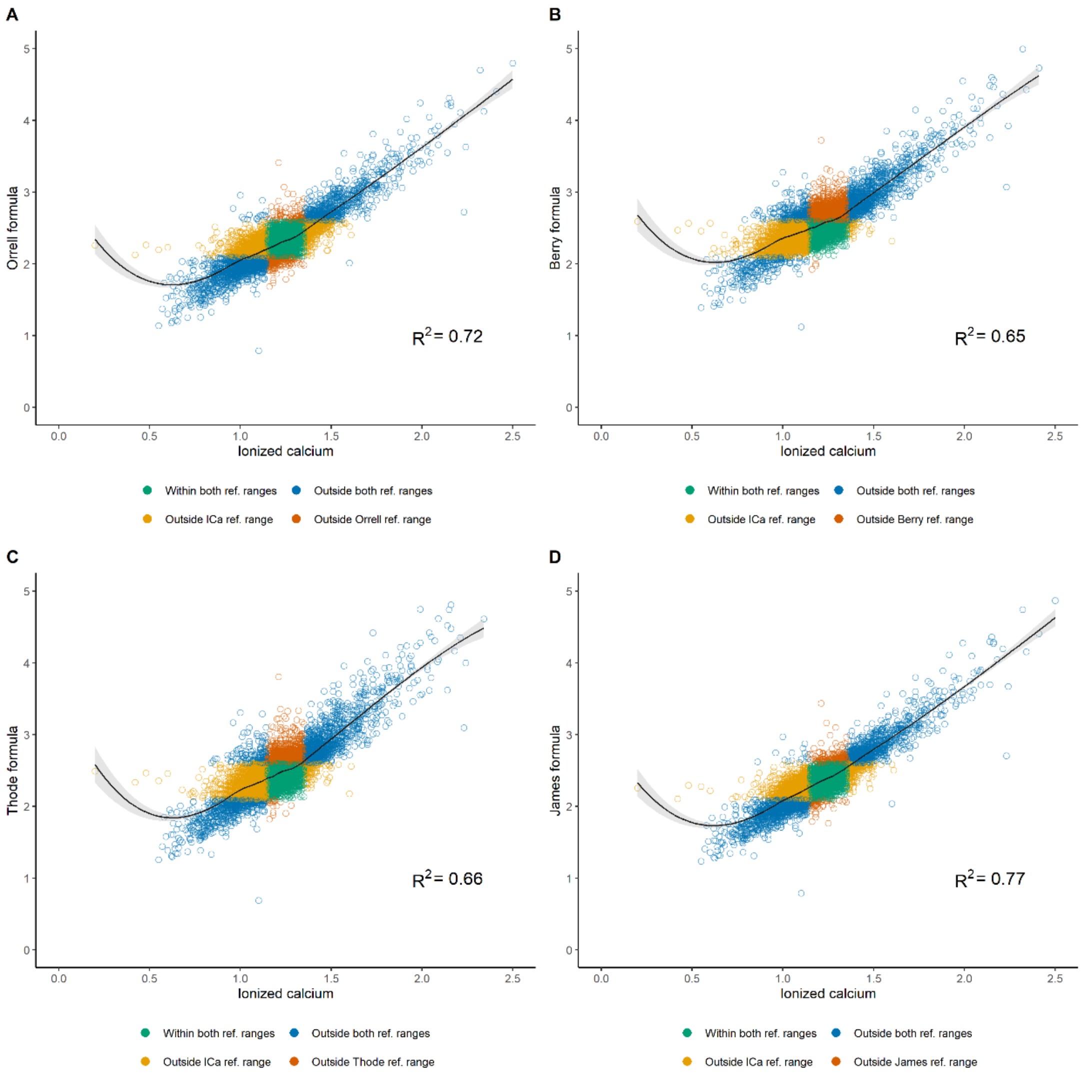

© 2025 Desgagnés N et al. *JAMA Network Open*.

eFigure 2: Correlation between total calcium and "adjusted" calcium (A = Antonio [A]; B = Antonio [B]; C = Antonio [C]; D = Pekar) vs. ionized calcium with corresponding correlation coefficient

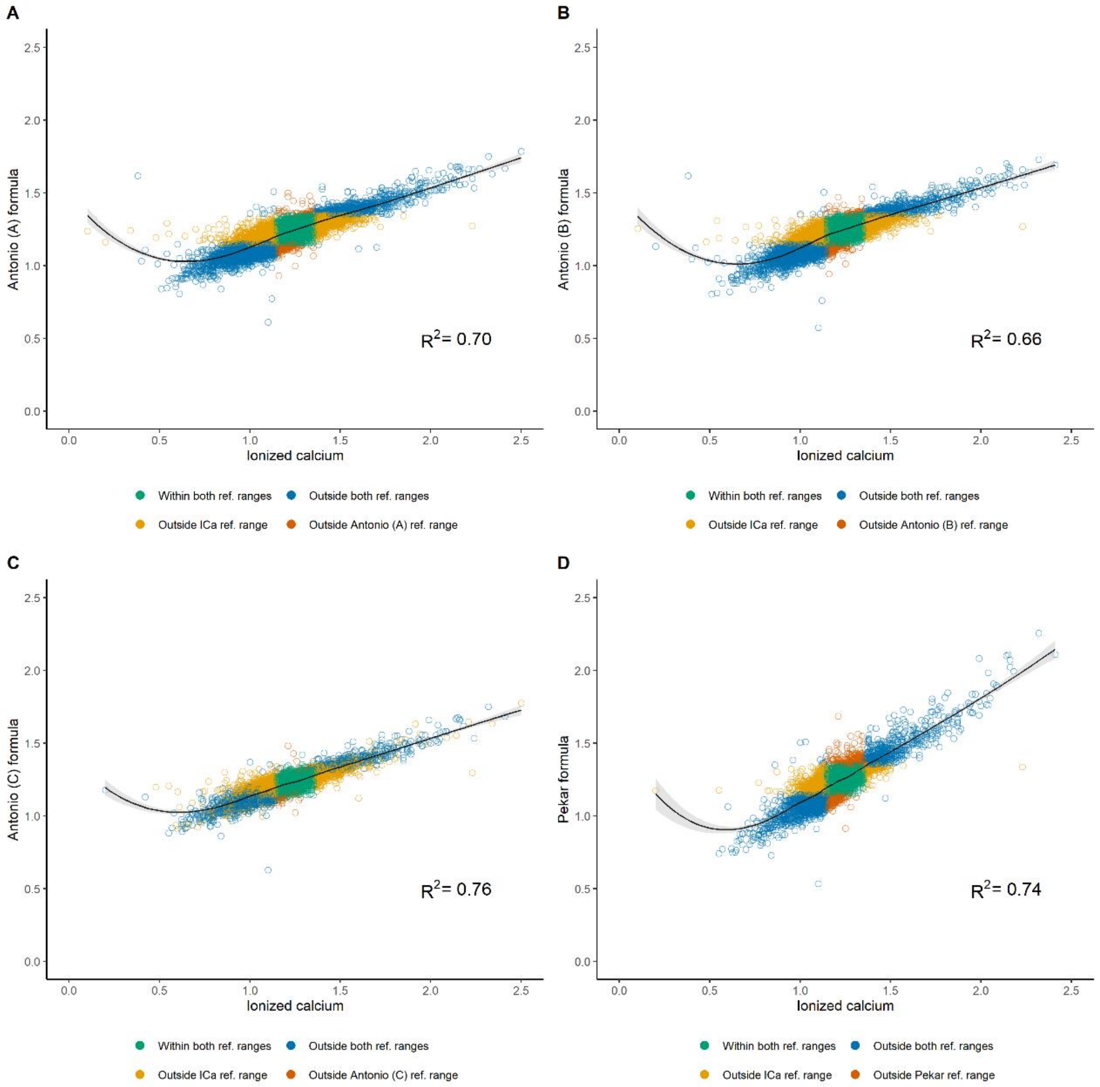

© 2025 Desgagnés N et al. *JAMA Network Open*.

eFigure 3: Bland-Altman Plot for total calcium (A), Payne (B), and Simplified (C) vs ionized calcium

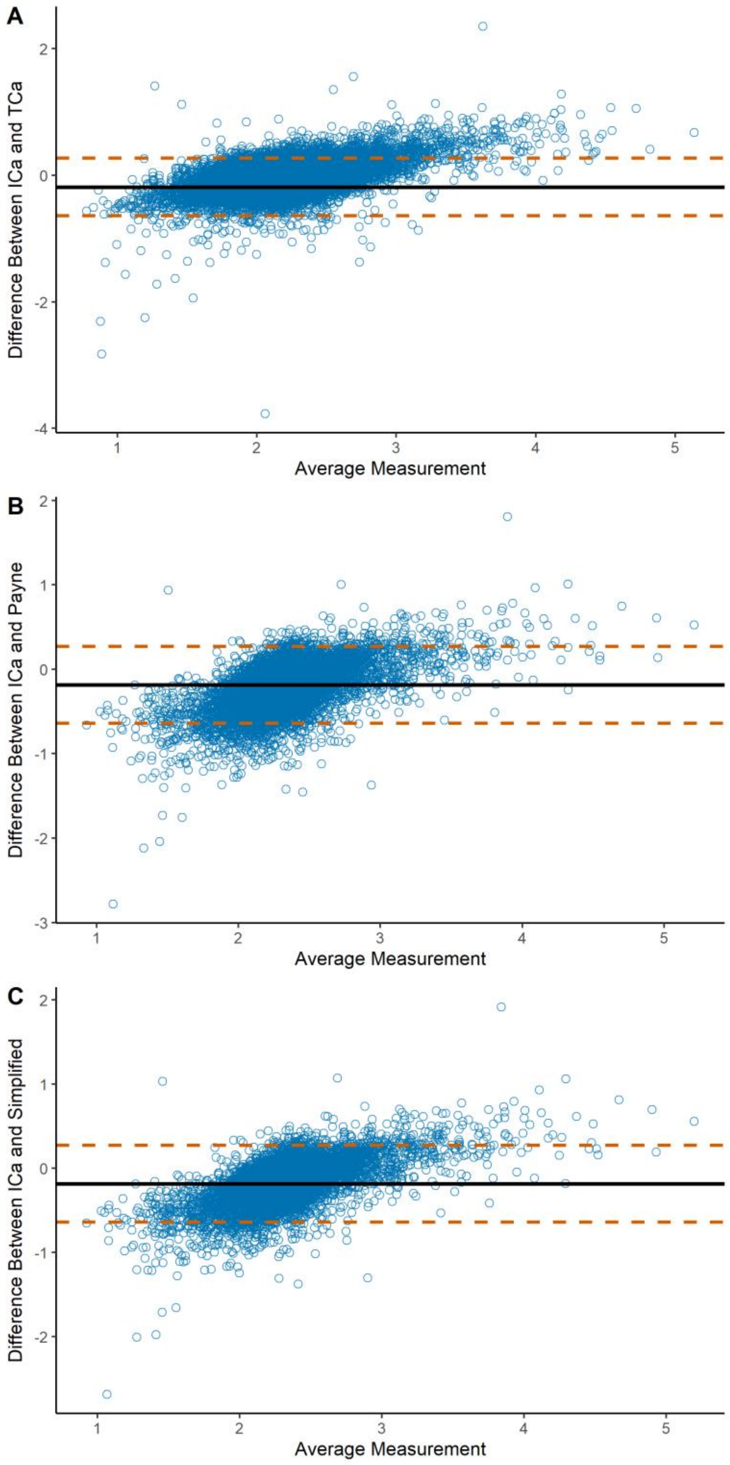

© 2025 Desgagnés N et al. *JAMA Network Open*.

eFigure 4: Bland-Altman Plot for Orrell (A), Berry (B), Thode (C), and James vs ionized calcium

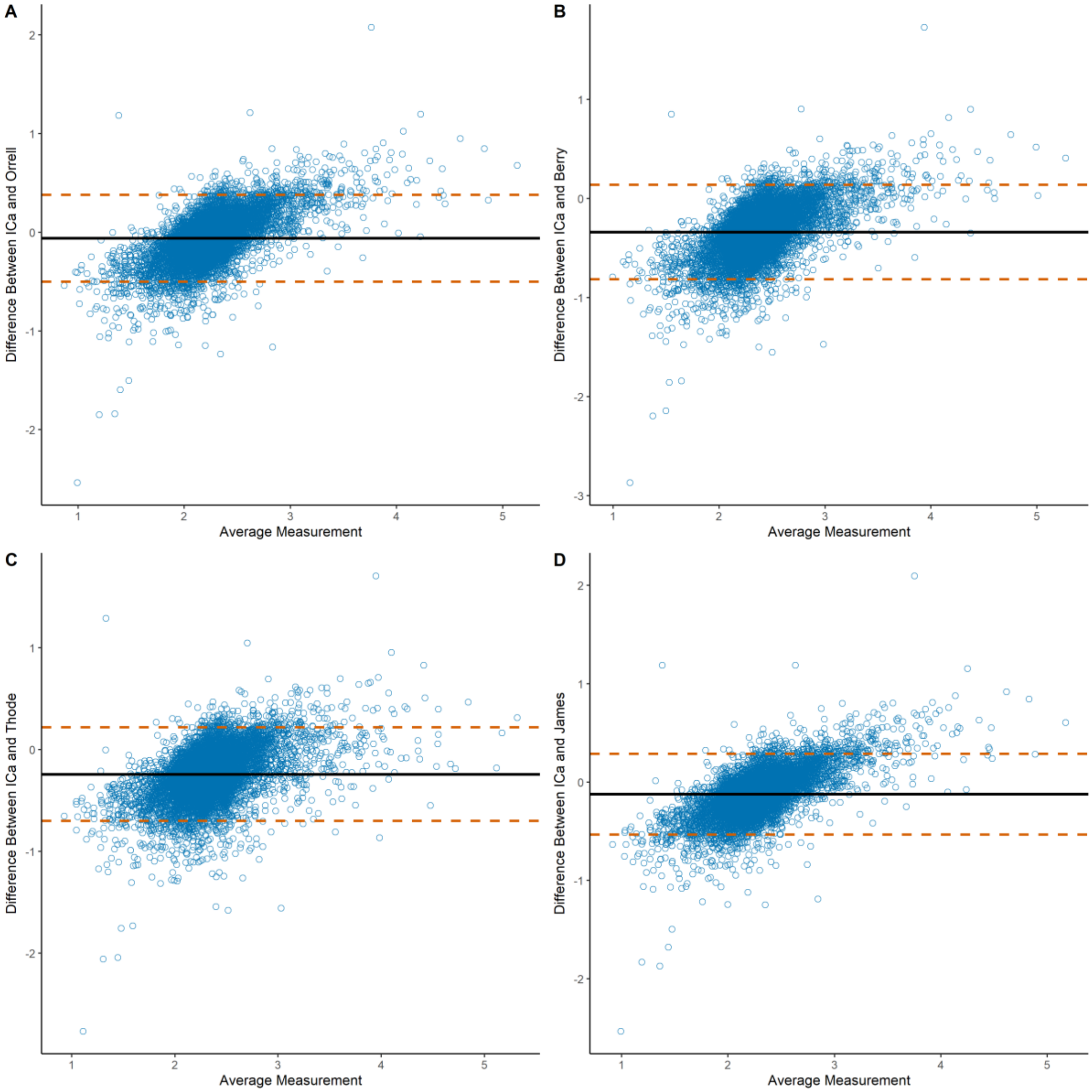

© 2025 Desgagnés N et al. *JAMA Network Open*.

eFigure 5: Bland-Altman Plot for Antonio A (A), Antonio B (B), Antonio C (C), and Pekar vs ionized calcium

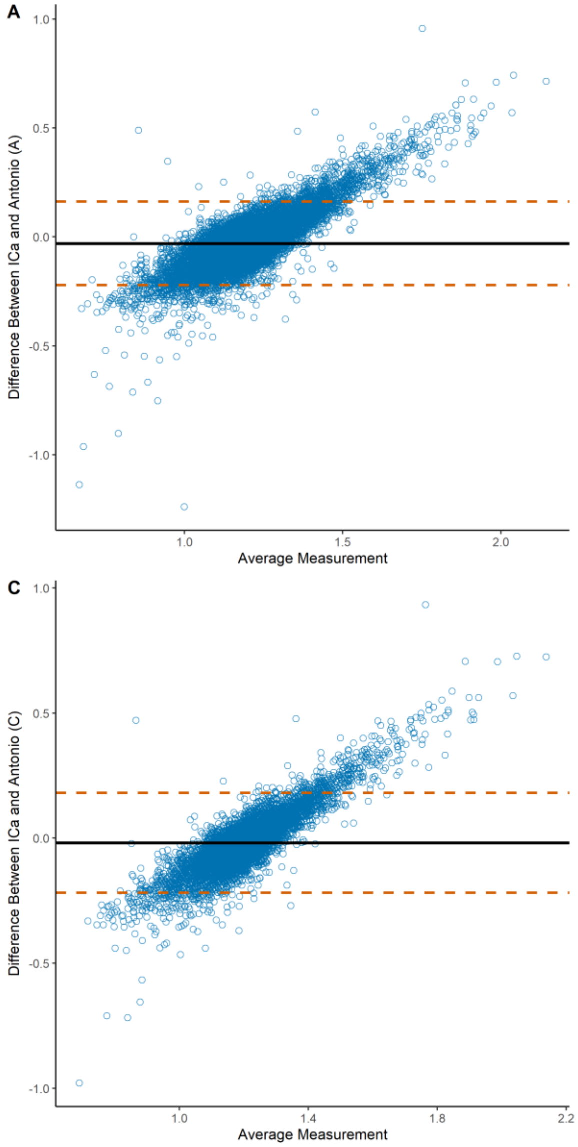

© 2025 Desgagnés N et al. *JAMA Network Open*.

eFigure 6. Number of total calcium and related tests ordered

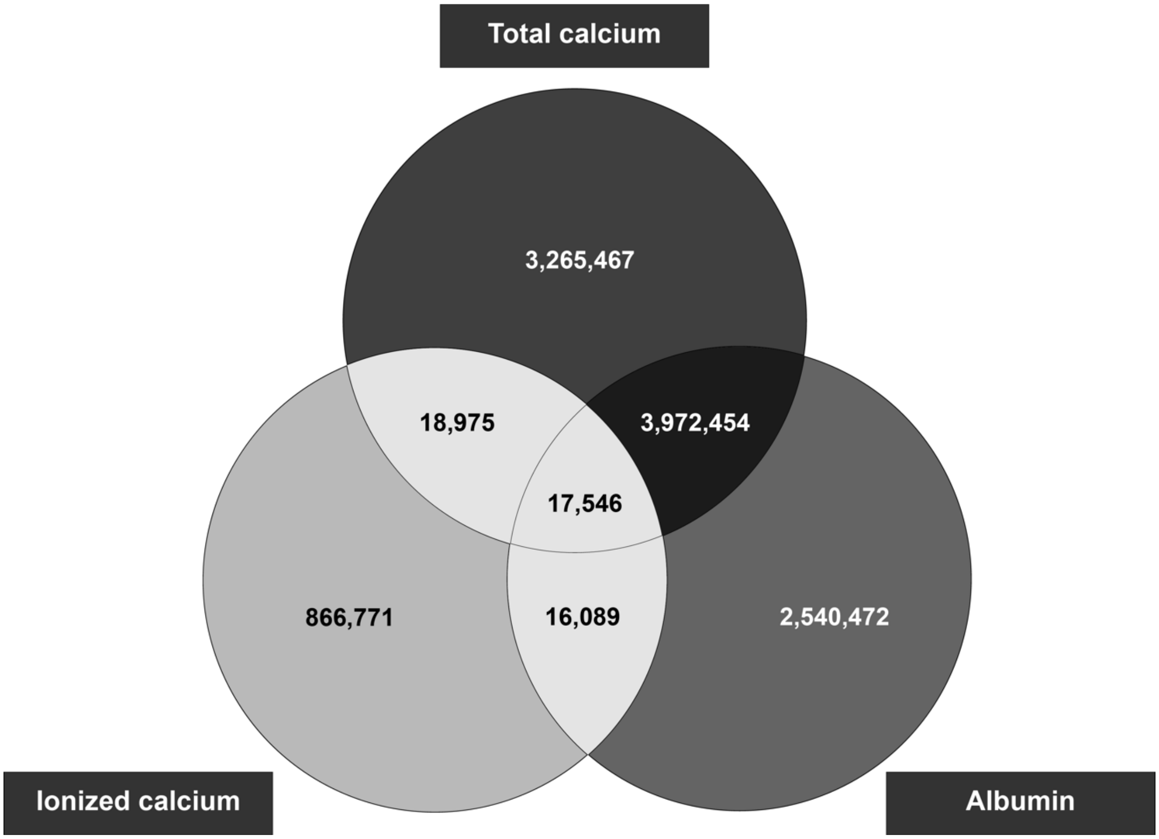

*Based on measurements collected simultaneously

© 2025 Desgagnés N et al. *JAMA Network Open*.

eFigure 7. Ordering pattern of total calcium, albumin, and ionized calcium over time

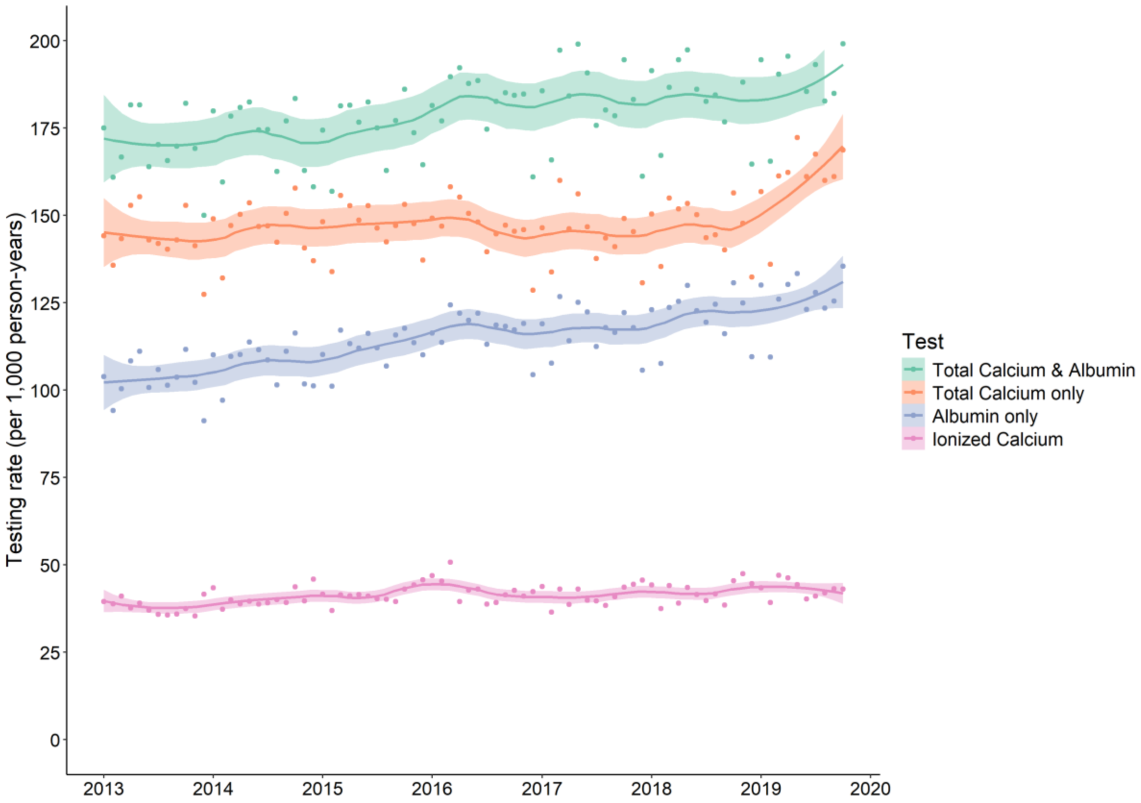

© 2025 Desgagnés N et al. *JAMA Network Open*.
