# Supplementary material for: Use of Albumin-Adjusted Calcium Measurements in Clinical Practice
Source: JAMA Netw Open. 2025 Jan 21;8(1):e2455251. doi: 10.1001/jamanetworkopen.2024.55251 (PMC11751745; doi:10.1001/jamanetworkopen.2024.55251)
Supplement: Supplement 2. — Data Sharing Statement [file jamanetwopen-e2455251-s002.pdf]

Data Sharing Statement

Desgagnés. Use of Albumin-Adjusted Calcium in Clinical Practice. *JAMA Netw Open*. Published January 21, 2025. doi:10.1001/jamanetworkopen.2024.55251

Data
Data available: No

Additional Information
Explanation for why data not available: The data from the present study are held securely in coded form within Alberta Health Services/Alberta Strategy for Patient Oriented Research Support Unit (AbSPORU) Data and Research Services team. Legal data-sharing agreements between AbSPORU and the data providers (e.g., health care organizations and government) prohibit AbSPORU from making the data set publicly available.
